# Polyomavirus infection: can early diagnosis prevent development of associated allograft nephropathy?

**DOI:** 10.1590/1678-4685-JBN-3986

**Published:** 2018-04-09

**Authors:** Marilda Mazzali

**Affiliations:** 1 Universidade Estadual de Campinas, Faculdade de Ciências Médicas, Laboratório de Investigação em Transplante, Campinas, SP, Brasil

Polyomavirus allograft nephropathy (PVAN) remains a diagnostic and therapeutic challenge in renal transplant recipients, being the main infectious cause of graft loss.^1^ Present immunosuppressive regimens are associated with about 40% prevalence of viruria followed by viremia from 5 to 30%. However, as the most severe infection case, polyomavirus allograft nephropathy occurs in 1 to 10% of patients and is associated with reduced allograft survival. In the first reports, PVAN was associated with a high risk of graft loss (close to 100%), but the majority of these studies were based on biopsy findings of viral inclusion in tubular cells, mostly in cortical areas. In 2000, Nickeleit et al described the clinical course of polyomavirus infection, showing that the presence of BKV DNA in plasma was a sensitive and specific method for identifying PVAN.^2^ Following that study, efforts were made to develop more specific, sensitive, and non-invasive methods for early detection of polyomavirus infection, including viral load in blood, plasma, and urine, detection of specific proteins (VP-1 PCR), quantitative urinary polyomavirus-Haufen testing, and polyomavirus genotyping.^1^

Early diagnosis of polyomavirus infection was associated with early therapeutic intervention, consisting mainly in the reduction in immunosuppressive therapy, alone or associated with antiviral protocols such as leflunomide or cidofovir. This approach was associated with an improvement in graft survival in cases where viruria or viremia cleared with therapy. However, in about 50% of cases, viremia persisted despite changes in immunosuppression, with a progression to graft loss. In addition, some patients developed acute rejection episodes within 6 months after PVAN diagnosis. Several studies aimed to correlate high plasma viral load with more severe allograft histology, classified as PVAN stage B and characterized by viral inclusions in cortical tubular cells and moderate interstitial inflammation. One of the hypothesis tested was that patients with presumed PVAN, i.e. absence of viral inclusions in renal biopsy, would have a lower viral load compared to patients with classical PVAN, with viral inclusions in medullar or cortical renal areas. Drachemberg et al. analyzed a series of sequential renal biopsies with presumed or classical PVAN and compared with BK viremia. The authors observed that despite comparable early viremia (no difference in BKV log between groups), persistence of viremia was associated with a worse allograft histology and graft loss. However, the authors were not able to determine a discriminative cutoff value for viremia associated with a viral clearance or viral disease progression.^3^

Transplant guidelines recommend polyomavirus infection screening with monthly quantitative plasma viral load for the first 3 to 6 months after transplant, then every 3 months until the end of the first post-transplant year; screening should also be performed whenever there is an unexplained rise in serum creatinine and after treatment of acute rejection. Reduction in immunosuppressive therapy is suggested for persistent plasma viral load ≥ 10.000 copies/mL.^4^ However, other groups suggest a lower viral threshold for patients with high risk.

The main disadvantage with polyomavirus DNA monitoring is the inter-assay variability, which complicates the interpretation of results and delays the intervention, with a negative impact on patient care. Many factors can be associated with this variability, including different standards, different primer designs, and genotypic BKV variation. In order to establish a common calibrator for plasma BK assays, the World Health Organization released the first International Standard for BKV, set at 7.2 log_10_ IU/mL. The majority of the commercial tests have been calibrated with the WHO standard, reducing the variation in BKV diagnosis.^4^^,^ 5

Godinho Pinto et al. compared an *in-house* polyomavirus detection assay with a commercial detection kit.^6^ The *in-house* test was directed against the VP-1 polyoma protein, considered the most stable viral area. Despite the difference in thresholds between detection assays, with a 2-log higher cutoff for the *in house* test, the efficacy for early detection of polyomavirus infection were comparable, with a satisfactory linear correlation (R^2^ = 0.83) between tests. 

The major contribution of the above paper is the development of a feasible and reproducible test for polyomavirus infection. The methods are clearly described and can be replicated by other groups, permitting early diagnosis and therapeutic intervention, with lower direct and indirect costs to transplant programs.
